# Funeral and Mortuary Operators: The Role of Stigma, Incivility, Work Meaningfulness and Work–Family Relation to Explain Occupational Burnout

**DOI:** 10.3390/ijerph18136691

**Published:** 2021-06-22

**Authors:** Gloria Guidetti, Annalisa Grandi, Daniela Converso, Nicoletta Bosco, Stefania Fantinelli, Margherita Zito, Lara Colombo

**Affiliations:** 1 Department of Psychological, Health and Territorial Sciences, University G. d’Annunzio of Chieti-Pescara, Via dei Vestini 31, 66100 Chieti, Italy; gloria.guidetti@unich.it (G.G.); stefania.fantinelli@unich.it (S.F.); 2 Department of Psychology, University of Turin, Via Verdi 10, 10124 Turin, Italy; daniela.converso@unito.it (D.C.); lara.colombo@unito.it (L.C.); 3 Department of Cultures, Politics and Society, University of Turin, Lungo Dora Siena 100/A, 10124 Turin, Italy; nicoletta.bosco@unito.it; 4 Department of Business, Law, Economics and Consumer Behaviour “Carlo A. Ricciardi”, Università IULM, Via Carlo Bo 1, 20143 Milan, Italy; margherita.zito@iulm.it

**Keywords:** funeral operators, burnout, stigma consciousness, meaningfulness of work, work–family spillover, supervisor incivility

## Abstract

The funeral and mortuary sector, including funeral homes, cemeteries and crematoria, is a largely neglected sector in regard to the study of occupational factors that can affect the quality of working life. The present study aimed at overcoming this gap by investigating job demands and resources that may affect burnout levels. Data were collected through a self-report questionnaire involving funeral industry employees (N = 229) from cemetery, morgues, crematoria and funeral agencies in a Northern Italian region. The survey was cross-sectional and non-randomized. Results reveal that among job demands, stigma consciousness, supervisor incivility and work-to-family negative spillover significantly affect levels of burnout, whereas meaningfulness of work and family-to-work positive spillover may represent relevant resources to counter the onset of burnout. The results of this study contribute to new insights into the psychosocial working conditions that affect occupational wellbeing among the funeral industry sector by also giving insight into how to promote resources to prevent burnout.

## 1. Introduction

The funeral and mortuary sector, including funeral homes, cemeteries and crematoria, is a largely neglected sector in regard to the study of occupational factors that can affect the quality of working life and mental health [1]. Unlike other occupational categories, such as health care and social workers, firefighters and police officers, who are exposed to death and suffering, funeral and mortuary workers are very often confronted with the social invisibility that permeates these professions [2], reflecting the widespread social taboos that surround death and the manipulation of dead bodies [3,4]. Despite this, workers in the funeral and mortuary sector constitute an essential professional category that must deal every day with work-related risks and job demands that may affect health and wellbeing. Above the most common job characteristics, such as the workload, the degree of autonomy and control, this occupational group has to confront work-related aspects such as counseling bereaved families, working with human remains and work-related social stereotypes [5,6,7]. 

Among the few studies that have been interested in assessing the quality of working life of this peculiar working population, only a small number have assessed how the psychosocial factors of the funeral context can specifically impact burnout levels [8]. This is surprising since, among other mental health issues such as depression, death anxiety and post- and secondary-traumatic stress disorder that have been more extensively analyzed [9,10,11,12,13,14], funeral and mortuary workers, due to their work environment, are more likely to suffer from compassion fatigue and burnout [15,16,17], which in turn may leave to deleterious consequences for their health-related behaviors and impaired work ability [1,15]. This would be in line with the abovementioned health and care professions, which have in common these suffering dynamics in their daily working life. However, traditional health and care professions are characterized by social visibility and prestige, contrary to cemetery and mortuary workers. Therefore, detecting well-known variables linked to depletion, distress and burnout would be conducive to better understanding this specific category.

Burnout is defined as a response to chronic work-related stress, the symptoms of which are defined as the experience of emotional exhaustion, cynicism (also known as depersonalization) and diminished professional accomplishment [18], where emotional exhaustion and cynicism are considered the two core dimensions [19]. Indeed, since reduced personal accomplishment refers to feelings of decline in one’s competence and productivity, and to one’s lowered sense of efficacy, representing the self-evaluation component of burnout, emotional exhaustion refers to feelings of fatigue and being depleted of emotional resources. Cynicism describes where employees develop cold, indifferent attitudes towards their job, coworkers and organization. 

Within the Job Demands–Resources (JD-R) model perspective [20,21], job demands, which refer to those physical, psychological, social or organizational aspects of the job with sustained physical and/or psychological (i.e., cognitive or emotional) impact, are expected to affect burnout levels [22,23]. Moreover, the JD-R model takes into account the role of job resources, which refer to those physical, psychological, social, or organizational aspects of the job that may reduce the physiological and psychological costs associated with job demands and negatively affect burnout levels [22]. According to the JD-R model, since every occupation has its own specific job demands and resources, the present study considered those that may be relevant to the funeral and mortuary occupational context to explain levels of emotional exhaustion and cynicism. 

Following the JD-R model as a framework, the aim of this study was to respond to the need to increase research and knowledge on this overlooked working population by considering both job demands and resources in relation to burnout levels. To this end, the findings will shed a light on how to deal with job-related psychosocial demands that are specific of the funeral industry sector as well as the role of resources that should be reinforced along with health-promoting guidance strategies targeted to this occupational population.

### Job Demands and Job Resources in the Funeral and Mortuary Occupational Sector

Workers such as those in the funeral and mortuary sector are constantly facing death and suffering. This peculiar aspect may imply both specific physical and psychosocial risks. On the one hand, studies have primarily focused on the role of noxious biological and physical risks implied in manipulating and exposure to pathogens of dead bodies [24,25] as well as the use of safety procedures to prevent physical hazards [26]. On the other hand, psychological and societal consequences should also be considered. The frequent and cumulative exposure to death, critical incidents as well as bereaved relatives and friends of the deceased may indeed affect levels of stress, lead to negative psychological changes and lower mental health [8,10,27]. All these elements may represent potentially traumatic events and involve high emotional labor [28] since workers are required to show compassion and display emotions according to the specific suffering situation [29]. The constant exposure to death has also significant implications of feeling stigmatized and socially isolated. 

In Western societies avoiding death is a way to control the fear of dirtiness and pollution associated with dead bodies [30]. Due to these physically tainting characteristics, these occupations have also been referred to as “dirty work” since they evoke some sense of rejection and repugnance [31], often associated with the widespread use of unconscious defenses [32]. Therefore, funeral and mortuary staff are often the object of stigmatization [2,3,33,34,35], a process that defines how people are socially discredited and disqualified [36] and that may threaten the way people define their own identity and attitudes of others towards employees in this sector. Pinel defined stigma consciousness as the extent to which people report attending to their stereotyped status [37], whereas occupational stigma consciousness is the extent to which employees are aware of the stigmatized nature of their job and believe that others treat them negatively because of it [38]. Since stigma consciousness is known to impair mental and physical health [39], in the same vein stigma consciousness derived from occupational membership may have negative consequences for both individual and organizational wellbeing. Previous studies have found occupational stigma consciousness to be predictive of increasing levels of burnout and deviant organizational production behaviors among blue and white collar workers and among call center employees [40,41]. Along this line of research, and according to the JD-R model [20,21], stigma consciousness may therefore be considered as a relevant job demand in affecting levels of emotional exhaustion and cynicism among the well-known stigmatized occupational group of funeral and mortuary workers. We thus state the following hypothesis:

**Hypothesis 1** **(H1).** *Stigma consciousness is positively related to emotional exhaustion (H1a) and cynicism (H1b).*

In considering the role of stigma consciousness, another relevant factor is the way by which people make use of strategies to deal with it. Following Ashforth and Kreiner [31], resignification represents the primary form of manipulation of work stigma. Through resignification people transform the professional social meaning, infusing positive values to the profession’s identity or refusing negative values [3], therefore increasing occupational identification and meaningfulness of work. Specifically, meaningfulness of work, which refers to the degree to which an employee finds his or her job meaningful, valuable and worthwhile [42], is an individual’s subjective sense of one’s job-related activities that are perceived as congruent with one’s personal values [43]. For workers in the funeral and mortuary sector, feeling that their work is full of meaning and useful, not only in utilitarian terms but also in social terms, can therefore constitute a significant job resource. This is also applicable to other helping professions [44] with risk of burnout. Thus, the following hypothesis was formulated:

**Hypothesis 2** **(H2).** *Meaningfulness of work is negatively related to emotional exhaustion (H2a) and cynicism (H2b).*

Along with stigma consciousness and meaningfulness of work, other central features that should be considered relating to wellbeing in such a working context is the relational dimension and the role of social support. Workers of the funeral and mortuary sector often produce group and work cultures based on complicity, solidarity and on the production of jokes and funny stories regarding their work as forms of renegotiation and resignification of their work identity [4,45,46] especially when faced with emotionally charged situations [47]. Since group cohesion is a fundamental social resource in the context of dirty work, it has also been noted that the role of supervisor support is a salient psychosocial factor that can help reconcile positive occupational identities in the face of continuous exposure to dirty tasks [48,49] as well as preserve functional personal resources such as work ability [1]. In this vein, among helping professions, it has been evidenced that the supervisor is the most important source of social support in relation to burnout dimensions [50,51]. 

On the other hand, supervisors in funeral and mortuary contexts are also perceived as outsiders, distanced and out of touch with the employees [3]. Indeed, as evidenced from Ashforth and colleagues [48], managers and supervisors in dirty work occupations may experience an identity dilemma between the shield status of managers and the threat status of being a member of a stigmatized occupation. An implication that can derive from these attitudes is therefore supervisor incivility, a source of workplace incivility. Workplace incivility has been defined as low-intensity deviant workplace behavior with an ambiguous intent to harm [52]. Estimates suggest that about 98 percent of employees are exposed to workplace incivility, which is recognized as a problem worldwide affecting employees in a wide variety of jobs and professions [53]. Among other sources of incivility behaviors, including from coworkers or customers, those deriving from the supervisor have been evidenced to be more harmful for employees’ and organizational consequences [53]. For example, due to supervisor incivility, emotional exhaustion may arise since employees undergo stress trying to suppress their negative emotions and thus negatively impacting intrinsic motivation and job performance [54]. 

According to these lines of research, we aimed at evaluating the role of both sides of the coin, that is to say, the role of employees’ perception of supervisor support and of supervisor incivility. In this vein, following the JD-R model [20,21], it is expected that supervisor support may act as a relevant job resource and supervisor incivility as a job demand respectively negatively and positively relate to emotional exhaustion and cynicism. In other words, we state the following hypotheses:

**Hypothesis 3** **(H3).** *Supervisor support is negatively related to emotional exhaustion (H3a) and cynicism (H3b).*

**Hypothesis 4** **(H4).** *Supervisor incivility is positively related to emotional exhaustion (H4a) and cynicism (H4b).*

Finally, in addition those demands and resources that originate within the working context, attention should also be paid to how the interface between work and family life may affect wellbeing of this occupational category. In the literature devoted to the study of the work–family interface, the emergence of conflict, or negative spillover, has primarily been considered between work and family domains. According to the role stress theory, negative spillover originates when one role interferes with or negatively impacts the participation in the other domain [55]. On the other hand, it has also been argued that being engaged in multiple roles can provide a greater number of opportunities for enriching experiences, such as income, opportunities for social support and heightened self-esteem and success, thus positively affecting wellbeing [56]. Therefore, through a positive spillover, the participation in one role may positively affect the participation in the other role. 

Although both negative and positive spillover may originate from both work and family domains, for the purposes of the present study, only negative work-to-family spillover and positive family-to-work spillover were considered. Specifically, due to the content of funeral and mortuary jobs, it is expected that negative spillover effects on work–family relations, in terms of strain and negative emotion originating in the work domain, may have significant consequences for the quality of personal and family life. As was pointed out by some authors, funeral and mortuary work can be considered as form of “emotional dirty work” as they “require the emotions of self and others to be managed in the face of feelings of loss, grief, guilt, conflict and even disgust” [34] (p. 701). According to previous studies, there are good reasons to consider workers involved in frequent and consistent emotional labor at work to be at higher risk of experiencing work-to-family interference [57,58], causing the employee to suffer from tension, fatigue and irritability, which ultimately affects family life [59]. Previous studies have also consistently shown that negative work-to-family spillover tends to occur more frequently than the opposite direction [60,61], which can significantly impact quality of working life, lessen job satisfaction and foster levels of burnout [57,58,62]. We thus state the following hypothesis:

**Hypothesis 5** **(H5).** *Negative work-to-family spillover is positively related to emotional exhaustion (H5a) and cynicism (H5b).*

Among the sources of positive family-to-work spillover, Grzywacz and Marks [63] recognized the role of support experienced in the family domain. As well as other sources of social support, those experienced within the family context may also positively affect wellbeing [63,64]. Recently it has been evidenced that emotional support experienced in the family domain is positively related to work satisfaction and negatively to work stress by fostering work–family balance in SME owners [65]. Therefore, for those who perform stigmatized “dirty work” and have to deal with death and suffering job-related contents, perceiving support from significant others outside the working context should be of value. Due to the family support, a positive family-to-work spillover effect is expected to be a relevant resource for funeral and mortuary workers by negatively relating to emotional exhaustion and cynicism. The following hypothesis was thus tested:

**Hypothesis 6** **(H6).** *Positive family-to-work spillover is negatively related to emotional exhaustion (H6a) and cynicism (H6b).*

## 2. Methods

### 2.1. Participants and Procedure

The data relied on in this study were collected as part of a research project aimed at assessing working life quality and psychosocial risks in funeral industry operators. Several premises located in a metropolitan area in Northwest Italy and involved in funeral work were contacted: 5 funeral services providers/supplies, 10 funeral agencies, 8 morgues, 1 multi-services cooperative, 3 crematoria and 1 citizen cemetery service. In Italy, not all funeral agencies are autonomous with regard to the provision of services. Some agencies therefore outsource some services (e.g., coffins, pallbearers, hearses, etc.) to larger funeral service companies (centri servizi in Italian). All but one of the companies (a funeral service provider/supplier) agreed to take part in the research and let the researchers administer the self-report questionnaires to the workers on duty. 

A cross-sectional design was used to collect data through a self-report questionnaire, which was administered during working hours. The research conforms to the Declaration of Helsinki of 1995 (and following revisions), and all ethical guidelines were followed as required for conducting human research, including adherence to legal requirements of the study country. Participants gave their informed consent prior to participating in the research session and agreed to anonymously complete the questionnaire. No treatment, including medical, invasive diagnostics or procedures causing psychological or social discomfort, was administered to the participants. Additional ethical approval was obtained by the bioethics committee of the University of Turin. 

In total, 260 employees agreed to filling in the questionnaire. After deleting questionnaires with at least one missing value, 229 questionnaires were correctly filled out and used for the present study. Of those, a total of 90 employees were females (39%) and 139 were males (61%) with a mean age of 43.6 (SD = 11.5). Most of the participants were full-time employees (86.7) with a mean length of service of 11.3 years (SD = 7.8). Regarding the service sector, 60 participants were employed in cemetery service, 43 in morgues, 43 in crematoria and 83 in funeral agencies. Of the sample, 69.1% stated that they had daily interaction with bereaved clients, 52.6% viewed corpses and 32.7% of the sample manipulated corpses on a daily basis.

Most of the participants were married (N = 114, 49.8%), whereas 35.4% (N = 81) were single/celibate and 11.8% were separated or divorced (N = 27). A small proportion (2.2%) were widows or widowers.

### 2.2. Measures

Stigma (ST) was measured using three items adapted from Wahl [66] (e.g., I worry that others will view me unfavorably because of my work). The scale ranged from 0 (totally disagree) to 3 (totally agree). 

Meaningfulness of work (MW) was measured using four items from the Copenhagen Psychosocial Questionnaire [67] (e.g., I think that my work is meaningful). The responses were given on a four-point scale ranging from 0 (totally disagree) to 3 (totally agree). 

Supervisor incivility (SI) was measured using three items (e.g., My supervisor ignores me) from the straightforward incivility scale [68]. The responses were given on a seven-point scale ranging from 0 (never) to 6 (always).

Supervisor support (SS) was measured using 4 items (e.g., My supervisor is supportive in working difficulties) using the organizational support scale by Caplan and colleagues [69]. The scale ranged from 0 (never) to 3 (always). 

Work-to-family negative spillover (NSpW/F) and family-to-work positive spillover (PSpF/W) were assessed using strain-based NSpW/F (e.g., Stress at work makes me irritable at home) and emotional support PSpF/W (e.g., Talking with someone at home helps me deal with problems at work), respectively, with four and three items adapted from [63]. Responses were given on a four-point scale ranging from 0 (never) to 3 (always).

### 2.3. Outcomes

Emotional exhaustion (EE) and cynicism (CYN) were measured using the corresponding five-item subscales from the Maslach Burnout Inventory—General Survey (e.g., I feel emotionally drained by my work; I have become less enthusiastic about my work) [70,71]. Responses were given on a 7-point scale ranging from 0 (never) to 6 (always).

### 2.4. Control Variables

Gender, age and marital status were inserted as control variables, since the literature recognized them as possible confounders in the relationships under study [72,73,74]. Moreover, since our sample consisted of workers employed in various services operating in the funeral industry sector, we also controlled for each of the services involved in the present study (cemetery, hospital morgue, funeral agencies and crematoria). Finally, the frequency of handling and viewing corpses and interacting with bereaved clients/persons was measured using a 4-point scale ranging from 1 (never) to 4 (every day).

## 3. Data Analysis

Data analyses were performed using IBM SPSS, Version 26, and Mplus 8.

Before analyzing data, the validity and reliability of the scales were evaluated. In particular, the technique of confirmatory factor analysis (CFA) was used to assess the dimensionality of the scales. Then, for each scale of the questionnaire, internal consistency was assessed by the Cronbach’s alpha coefficient, and synthetic indexes were then calculated. 

After descriptive (mean [M] and standard deviation [SD]) analysis of each synthetic index, hierarchical multiple regression models were established to evaluate which demands and resources influenced funeral industry operators’ psychological health at work. We specified two separate regression models for the two dependent variables, emotional exhaustion and cynicism. In the hierarchical regression process, predictor variables are added in successive steps (enter method) based on their theoretical status. In the final step, control variables were inserted. This model estimation process allowed us to evaluate if, after adding new predictive variables, the predictors inserted in the later steps explained a significant portion of the variance over and above the variables inserted at the previous steps. Then, at each step, the holistic fit index, useful for evaluating the model’s solution quality (R^2^ coefficient), can increase (ΔR^2^), showing the marginal utility of the most recently added variables.

## 4. Results

First, two CFAs were conducted to compare an eight-factor model, one for each construct of this study, with a model in which all the items were grouped into a single dimension using an MLR estimator. The eight-factor model showed a greater fit to data (χ^2^ = 733.65; df = 406; χ^2^/df = 1.80; comparative fit index (CFI) = 0.91; Tucker–Lewis index (TLI) = 0.90; root mean square error of approximation (RMSEA) = 0.06, 90% CI 0.052–0.066; standardized root mean square residual (SRMR) = 0.06) compared to the model with a single factor grouping all the items (χ^2^ = 2218.29; df = 434; χ^2^/df = 5.11; CFI = 0.46; TLI = 0.41; RMSEA = 0.13, 90% CI 0.12–0.14; SRMR = 0.12). The fit values of the eight-factor model were good, and each item loaded into its factor with saturation values greater than 0.50.

ANOVAs were run in order to analyze if differences occurred between relevant sociodemographic variables such as gender, service sector and marital status, and correlations between variables were assessed. Significant differences in the study variables occurred between males and females regarding levels of stigma, which was significantly higher for males (F = 5.58, *p* = 0.019). Among service sector (cemetery, hospital morgue, funeral agencies and crematoria), cemetery employees were significantly lower for meaningfulness of work and significantly higher for supervisor incivility, work to family negative spillover, emotional exhaustion and cynicism compared to the other services (F_MW_ = 3.44, *p* = 0.018; F_IS_ = 5.57, *p* = 0.001; F_NSPWH_ = 5.58, *p* = 0.001; F_EE_ = 10.54, *p* = 0.000; F_CY_ = 12.57, *p* = 0.000). No significant differences were observed between marital statuses (married, single, separated/divorced and widowed). Therefore, marital status was not entered as a control variable in the final regression model.

Table 1 shows the results of the correlation analysis between all variables considered for the present study, followed by the means, standard deviations and Cronbach’s alphas. The scales and subscales had adequate internal consistency, and all the variables correlated in the expected direction. Surprisingly, the increasing frequency in viewing corpses was negatively related to NSpW/F, EE and age, whereas the increasing frequency in interacting with bereaved clients was negatively associated with cynicism and age. The increasing frequency in handling corpses was on the other hand positively associated with ST.

Table 2 shows the results of the multiple regression analysis. In the first model, job demand and resources regarding stigma consciousness (ST) and meaningfulness of work (MW) were entered. Concerning EE, ST had a significant and positive relationship, and MW showed a negative relationship. In the second model, variables regarding relationships in the work environment were entered. SI was significantly and positively related to EE. On the other hand, SS was not significantly associated with EE, and only MW remained significantly associated with EE. In the third step, variables concerning the interface between work and family in its positive and negative aspects were inserted. Specifically, NSpW/F was positively associated with EE, whereas PSpF/W was negatively and significantly associated with EE. At this step, MW stopped being significant. Finally, in the fourth step after inserting control variables, SI and NSpW/F still remained significantly and positively associated with EE, and PSpF/W remained negatively and significantly associated with EE. Among control variables, being a cemetery employee was significantly and positively associated with increase in EE. No significant changes occurred regarding relationships between independent variables and EE.

Concerning CY, in the first the regression model, ST had a significant and positive relationship, whereas MW showed a negative relationship with CY. In the second model, variables regarding relationships in the work environment were entered. SI was significantly and positively related to CY along with ST and MW. On the other hand, SS was not significantly associated with CY. In the third step, variables concerning the interface between work and family in its positive and negative aspects were inserted. Specifically, NSpW/F was positively associated with CY, whereas PSpF/W was not. At this step, ST, MW and SI were still significantly associated. Finally, in the fourth step after inserting control variables, no significant changes occurred regarding relationships between independent variables and CY. Among control variables, being a cemetery employee was significantly and positively associated with increase in CY. 

The change in R^2^ was significant for both the regression models with EE and with CY as dependent variables, increasing when adding the step two variables above the first step variables, and when adding the variables regarding the spillover between work and family variables. Otherwise, no increasing change in R^2^ was observed in the last step when inserting control variables.

## 5. Discussion

The aim of the present study was to help expand current research in regard to the context of funeral and mortuary jobs, since they are significantly overlooked especially regarding the study of workplace psychosocial factors that may affect wellbeing [1,72]. To achieve that goal, the JD-R model [20,21] was used as a theoretical framework as it accounts for both the role of job demands and of resources. This approach therefore has also practical implications since it can focus not only on job-related risk prevention strategies but also on which significant resources it is possible to invest and promote. Moreover, although the existing research previously identified psychosocial risks in the funeral and mortuary sector [5,8,15,16,72], there is a lack of studies that systematically evaluate both the most relevant job demands and resources and their relationship with the onset of the two core dimensions of burnout, which are emotional exhaustion and cynicism. Among other occupations, a wide array of literature has to date evidenced both health-related and organizational consequences of burnout, e.g., [74,75], that in the funeral context may specifically affect the quality of the service provided and the onset of behaviors that pose a risk to health. Indeed, being exhausted and with cynical attitudes towards one’s work may have deleterious effects on the quality of relationships with bereaved clients, lessening empathy and fostering counterproductive work behavior. Furthermore, poor mental health related to burnout may keep those workers from healthy behaviors and being more engaged in unhealthy habits such as alcohol consumption, substance use and poor weight management [15] by also limiting the possibility of maintaining adequate levels of work ability [1].

Among job demands, the present study considered the role of stigma consciousness (ST), supervisor incivility (SI) and negative work-to-family spillover (NSpW/F), whereas among job resources meaningfulness of work (MW), supervisor support (SS) and positive family-to-work spillover (PSpF/W) were considered. Hierarchical regression analyses were run in order to evaluate what demands and resources are most relevant in affecting levels of burnout among funeral and mortuary workers, and four different models were run. Regarding emotional exhaustion (EE) as the outcome variable, in terms of the first and second hypotheses, ST (H1a) and MW (H2a) stopped being significantly associated with EE after inserting relational, SI and SS and work–family interface variables. Indeed, despite SS not emerging as a significant predictor of EE, the final model evidenced that the most relevant factors were SI, NSpW/F and PSpF/W. Therefore, only H3a, H5a and H6a were confirmed. Regarding cynicism (CY) as the outcome variable, in terms of the first and second hypotheses, ST (H1b) and MW (H2b) were still significantly associated with CY after inserting relational and work–family interface variables. Indeed, although SS did not emerge as a significant predictor of CY, the final model evidenced that the most relevant factors were ST, MW, SI and NSpW/F but not PSpF/W. Therefore, H1b, H2b, H3b and H5b were confirmed.

Overall, these results are in line with the assumptions of the JD-R model [20,21] by highlighting the energy depleting process induced by job demands and on the other hand the protective role of job resources. Specifically, different features of funeral and mortuary jobs that may differently affect the emergence of either emotional exhaustion or cynicism are evident. Indeed, on the one hand it is possible to observe that SI and NSpW/F mostly affected both EE and CY, thus evidencing how these job demands represent relevant factors for this context. On the other hand, it was further highlighted that, if for the prediction of EE the emotional support experienced in the family context (PSpF/W) played a central role, for CY this central role was played by both ST and MW. Finally, contrary to our expectations about the negative relationship between supervisor support and EE and CY, no significant relationships emerged in this study. Among the control variables, workers employed in cemeteries, compared to those employed in funeral homes, morgues and crematoria, were the most vulnerable to reporting higher levels of burnout.

## 6. Limitations

The current findings should be considered while taking into account some limitations. First, the study involved a non-randomized sample of funeral and mortuary workers from a region in north of Italy; therefore, the generalization of results should be cautious. Future studies should improve this field of research by involving participants at several funeral and mortuary services of other Italian regions and possibly involving other countries. Moreover, the cross-sectional nature of this study does not allow causal inferences for relationships among the variables. Therefore, future research should enhance longitudinal studies through repeated administration of the instrument to analyze if reciprocal association between the study variables may emerge.

## 7. Conclusions

Despite these limitations, the results of this study should provide notable implications for the state of the art concerning work-related wellbeing in the funeral and mortuary sector. Due to the lack of studies related to this area, it is not possible to provide strong comparative evidence, but the first evidence and future study and research directions are given.

The evidence provided from this study highlights that among the most relevant psychosocial risks in affecting burnout in the context of funeral and mortuary jobs, there are supervisor incivility and work-to-family negative spillover. On the one hand it is possible to highlight that, although supervisor social support should play an important role in dirty work, especially because it can lead to a more positive occupational identity [45] and by prevent decrease in work ability [1], findings from this study showed no significant relationship with burnout dimensions when controlling for its negative counterpart, that is supervisor incivility. Indeed, behaviors of incivility experienced in the relationship with the supervisor had the greatest impact. On the one hand it can be outlined that supervisor support might exert a more significant impact on positive and organizational outcomes, which according to the JD-R model are sustained from the motivational process of work engagement [20,21]. In this vein, future research should focus also on positive outcomes. On the other hand, the role of supervisor incivility is consistent with previous findings that emerged in other occupational sectors [53], which may be read in the light of what has been outlined by Ashforth and Kreiner [31] in regard to dirty work. Following these authors [31], the identity dilemma with which superiors and managers in the context of dirty work often have to face can lead them to act with detachment towards their subordinates. Indeed, as further highlighted by the qualitative study of other scholars conducted among funeral directors [3], supervisors are often perceived as outsiders, distanced and out of touch with the employees, which may also spread into rude and demeaning attitudes. These findings therefore highlight how incivility represents a relevant psychosocial risk factor even for the funeral and mortuary sector, which has received, to date, little attention from scholars. Beyond the reasons that might favor these attitudes on the part of supervisors, an aspect that future research can examine from the perspective of the actor, these results allow us to underline how it should be necessary to provide intervention tools aimed at improving the quality of leadership and to prevent incivility. To this end, supervisors should be trained to model appropriate behaviors and social skills, demonstrating for example their trustworthiness and earning their subordinates’ trust [76], how to deal with their work identity dilemma and knowledge of the harmful effects of incivility. 

In regard to the result of the role of negative work-to-family spillover, the evidence emerged suggests that the strain experienced in funeral workplace contexts, which is mainly of an emotional nature [8,34], may easily spills over into the family domain. This is also consistent with what emerged from previous studies conducted in other contexts with a high emotional load [57,58], underlining the need to provide for preventive measures that limit these consequences. For example, as outlined by a narrative study [34], recovery experiences, in the form of psychological detachment, may help to release from the pressure and strain experienced in the funeral working context. This strategy may also provide a resource to cope with the emerging negative work-to-family spillover [77]. 

On the other hand, this study also highlights that the support experienced in the family environment lessens the levels of emotional exhaustion. This is consistent with previous studies [65], which also provided evidence for this relationship to be mediated by work–family balance. Although no previous studies, to the best of our knowledge, considered this kind of resource for funeral and mortuary workers, a recent work/life resource program provided from the National Funeral Directors Association [78] specifically outlined the relevance of supporting a funeral director in the family and how to sustain funeral workers in self-care behaviors. In this vein, as our study not only included funeral directors but also cemeteries and morgues employees, these results may directly inform practical guidelines in fostering such interventions among the funeral and mortuary professional categories. Moreover, programs based on the awareness on job risks and characteristics are also functional from a positive perspective in order to prevent the depletion of resources with negative consequences on individual balance and health [79].

Our findings also contributed in regard to the role of stigma consciousness. Although it is known that funeral and mortuary workers have to face and manage occupational stigma [3,4,33,34,35], few studies specifically evaluated if the awareness of the stigmatized nature of their job may increase burnout symptoms. In the light of the results that emerged from this study, it is possible to highlight how stigma consciousness is able to increase cynical and detached attitudes towards work. This supports a contribution in theoretical terms, as it expands the existing research that otherwise evidenced only the relationship with emotional exhaustion [40] and integrates the role of stigma as a job demand within the JD-R model [20,21]. On the other hand, it underlines how the awareness of belonging to a stigmatized working category can induce people to adopt defensive strategies for their identity and self-esteem. Cynicism has indeed been defined as both a coping strategy and a self-defensive attitude directed towards the employing organization to distance from stress and to restore dignity [80]. In this vein, cynicism may represent a set of attitudes adopted to restore self-image of those with the stigmatized occupational identity, though it may have deleterious effects for the organization and quality of work by negatively affecting prosocial behaviors and performance [80,81]. A valued job resource that on the contrary has emerged to negatively relate to cynicism is meaningfulness of work. This result is consistent with existing evidence regarding both the negative association between meaningfulness of work and depersonalization [44] as well as the lack of experienced meaning and its relationship with negative outcomes, and in particular employee cynicism [82]. In contrast to other resources, such as supervisor or family support, meaningfulness of work operates as a resource that favors an alignment between the self-image and the work identity [43]. Therefore, such an integration may be of value especially when belonging to a stigmatized occupation, preventing cynical self-defensive attitudes from being adopted. Moreover, the image of the job should be carefully managed, particularly in this specific profession. The perception of a negative social evaluation of work seems to be linked to burnout [83] and to other negative organizational outcomes such as counterproductive work behaviors, and moral disengagement, which has been widely studied between healthcare workers [84] and, in the funeral sector, can lead to non-ethical behaviors. These conclusions can therefore also have resonance in practical terms. Targeted interventions aimed at fostering meaningfulness of work should be applied. For example, it has been evidenced that trait mindfulness at work is able to sustain more authentic and true self-values and behaviors by fostering meaningfulness of work [44]. Therefore, among others, mindfulness-based interventions (MBIs) may work to help workers in funeral and mortuary sectors to go through a process of re-signifying the work identity in the face of stigma and to introduce self-care strategies that may be particularly helpful to deal with potentially traumatizing exposure to death and suffering [85].

In the light of these conclusions, managerial implications can be derived for prevention and health promotion programs. On the one hand, psychosocial risk and quality of working life assessment programs should be implemented that make it possible to periodically monitor the organizational health status and employee wellbeing. Furthermore, organizational management could intervene through, for example, secondary prevention strategies that directly involve those in supervisory roles. To this end, intervention programs developed to provide civil interaction at the workplace have helped in reducing incivility, absence and burnout and improving managerial trust and organizational commitment [86]. Initiatives that enhance work–life balance and recovery abilities and restore meaningfulness of work, through for example stress management interventions, should be encouraged, along with initiatives that promote wellbeing and health education involving workers’ families by promoting their roles as sources of social support.

## Figures and Tables

**Table 1:** Descriptive statistics and correlations among the study variables.

	1	2	3	4	5	6	7	8	9	10	11	12
1. ST	1	−0.092	0.197 **	−0.094	0.145 *	−0.188 **	0.153 *	0.231 **	−0.001	0.106	0.135 *	0.126
2. MW		1	−0.235 **	0.198 **	−0.280 **	0.118	−0.278 **	−0.431 **	0.136 *	0.056	0.057	0.159 *
3. SI			1	−0.461 **	0.367 **	−0.214 **	0.445 **	0.413 **	−0.018	−0.063	0.057	−0.053
4. SS				1	−0.157 *	0.121	−0.215 **	−0.280 **	0.06	0.007	−0.095	0.042
5. NSpW/F					1	−0.046	0.646 **	0.451 **	0.081	−0.189 **	−0.127	−0.026
6. PSpF/W						1	−0.255 **	−0.185 **	−0.074	−0.094	−0.037	−0.038
7. EE							1	0.636 **	0.086	−0.146 *	−0.09	−0.034
8.CY								1	0.05	−0.073	−0.067	−0.131 *
9. Age									1	−0.131 *	−0.079	−0.140 *
10. Handling corpses										1	0.746 **	0.400 **
11. Viewing corpses											1	0.358 **
12. Interaction with bereaved clients												1
Mean (SD)	2.33 (1.88)	8.72 (2.51)	3.15 (4.44)	7.52 (3.23)	3.75 (2.76)	6.31 (2.27)	11.24 (8.75)	7.45 (6.90)	43.62 (11.43)			
Cronbach’s α	0.70	0.82	0.88	0.84	0.87	0.84	0.91	0.78				
Range	0–9	0–12	0–9	0–12	0–12	0–9	0–30	0–30				

*p* < 0.05; * *p* < 0.01; ** *p* < 0.001. ST, stigma; MW, meaningfulness of work; SI, supervisor incivility; SS, supervisor support; NSpW/F, negative work–family spillover; PSpF/W, positive work–family spillover; EE, emotional exhaustion; CY, cynicism.

**Table 2:** Regression parameters: standardized coefficients and overall changes in R^2^ for emotional exhaustion and cynicism.

Model			EE		CYN
		Beta	SE	Beta	SE
1	ST	0.132 *	0.299	0.201 ***	0.217
	MW	−0.249 ***	0.225	−0.406 ***	0.164
	Adj R^2^	0.07		0.21	
	ΔR^2^	0.08 ***		0.22 ***	
2	ST	0.064	0.28	0.147 **	0.208
	MW	−0.163 ***	0.214	−0.333 ***	0.159
	SI	0.401 ***	0.134	0.277 ***	0.1
	SS	0.011	0.183	−0.068	0.136
	Adj R^2^	0.21		0.29	
	ΔR^2^	0.14 ***		0.09 ***	
3	ST	−0.004	0.232	0.116 **	0.203
	MW	−0.046	0.178	−0.276 ***	0.156
	SI	0.188 ***	0.116	0.175 ***	0.102
	SS	−0.015	0.149	−0.08	0.13
	NSpW/F	0.548 ***	0.169	0.269 ***	0.148
	PSpF/W	−0.183 ***	0.194	−0.076	0.17
	Adj R^2^	0.48		0.35	
	ΔR^2^	0.26 ***		0.06 ***	
4	ST	−0.002	0.238	0.202 **	0.147
	MW	−0.041	0.188	0.159 ***	−0.24
	SI	0.149 **	0.121	0.103 **	0.116
	SS	−0.033	0.152	0.129	−0.108
	NSpW/F	0.528 ***	0.175	0.148 ***	0.261
	PSpF/W	−0.175 ***	0.198	0.168	−0.052
	Age	0.006	0.042	0.036	−0.017
	Gender (Male = 1)	−0.021	1.073	0.91	−0.107
	Cemeteries	0.167 **	1.356	1.149 *	0.248
	Hospital Morgue	−0.024	1.451	1.23	0.012
	Crematoria	0.094	1.507	1.277	0.055
	Viewing Corpses	0.053	0.585	0.496	0.229
	Handling Corpses	0.013	0.539	0.457	−0.073
	Interaction With Bereaved People	0.018	0.504	0.427	−0.096
	Adj R^2^	0.48		0.39	
	ΔR^2^	0.020		0.06 **	

* *p* < 0.05; ** *p* <0.01; *** *p* < 0.001. ST, stigma; MW, meaningfulness of work; SI, supervisor incivility; SS, supervisor support; NSpW/F, negative work–family spillover; PSpF/W, positive work–family spillover; EE, emotional exhaustion; CY, cynicism; SE, standard error.

## Data Availability

The data presented in this study are available on request from the corresponding author. The data are not publicly available due to the Italian law on privacy.
